# Settling and Along-Isopycnal Subduction of Small Microplastics Into Subsurface Layers of the Western North Pacific Ocean

**DOI:** 10.1021/acs.est.5c08983

**Published:** 2025-09-18

**Authors:** Mao Kuroda, Atsuhiko Isobe, Keiichi Uchida, Ryuichi Hagita, Satoru Hamada

**Affiliations:** a Research Institute for Applied Mechanics, Kyushu University, 6-1 Kasuga-koen, Kasuga 816-8580, Japan; b 26412 Tokyo University of Marine Science and Technology, 4-5-7 Konan, Minato-ku, Tokyo 108-8477, Japan

**Keywords:** small microplastics, isopycnal transport, subsurface layer, subduction

## Abstract

The vertical distribution of small microplastics (SMPs; 10–300 μm in size) and its relation to water masses were investigated through seawater sampling and hydrographic surveys from the sea surface to 1000 m in the North Pacific Ocean. The average ± standard deviation of SMP concentrations in 12 layers at four stations was 6910 ± 2620 particles m^–3^. Concentrations were high in isopycnal layers between potential densities of 23 and 25σ_θ_ (100–300 m depths). Elevated concentrations were also frequently detected below the North Pacific Intermediate Water (NPIW), characterized by a salinity minimum around the 26.6–27.0σ_θ_ (approximately 600 m depth) isopycnal layers. A simple modeling approach to reproduce the observed SMP distribution suggested two pathways for SMPs floating in surface convergence zones. One pathway is the weak settling of SMPs of which the density becomes close to neutral, causing the along-isopycnal subduction from isopycnal layers outcropping at the sea surface to subsurface layers above the NPIW. Therefore, the global inventory of weakly settling near-neutral SMPs is expected to be high in the subsurface layers. Meanwhile, the strong settling via biological processes causes the other pathway from the surface euphotic layer to deep layers that never outcrop at the sea surface.

## Graphical abstract

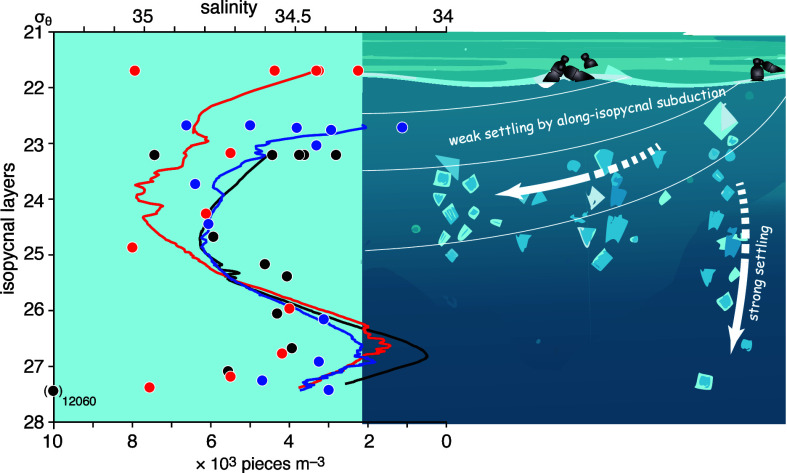

## 1 Introduction

Small microplastics (SMPs), which is defined as microplastics (MPs) smaller than 300 μm, are likely to be abundant in ocean subsurface layers as well as the sea surface because less-buoyant plastic particles easily move downward due to both oceanic turbulence and settling via fecal pellets excreted by zooplankton,^1–3^ absorption into marine aggregates,^4–8^ and biofouling by algae.^9,10^ However, there is currently no straightforward method to evaluate the presence of SMPs in the actual ocean, as standard protocols have not been established for sampling particles invisible to the naked eye or for laboratory analyses that minimize contamination, particle loss, and further potential fragmentation of fragile, degraded plastic particles. To the best of our knowledge, the first research to report pelagic SMPs dates to the mid 2010s, when SMPs drifting several meters below the sea surface were collected via the seawater intake of a vessel in the North Pacific and Atlantic Oceans (Table S1).^11,12^ Surface sampling using nets with fine mesh sizes has also been capable of detecting SMPs in the surface layer.^13,14^ Sampling of seawater containing SMPs using bottle samplers or water pumps has been useful for collecting SMPs drifting in layers shallower than several hundreds of meters from the sea surface.^15–17^ Efforts extending to abyssal oceans (>1000 m) have detected SMPs in the Arctic Ocean,^18^ South Atlantic Ocean,^19^ and North Pacific Ocean,^20,21^ using a pump system (large volume water transfer system; WTS-LV) to transfer a large volume of seawater through a steel filter at predetermined depths. Such pioneering studies uncovered SMP concentrations (particle count per unit seawater volume) in the abyssal ocean ranging from 0 to >1000 particles m^–3^, comparable to concentrations observed in the upper oceans (Table S1).

We consider how vertical profiles of SMPs are determined in the open ocean. The abundance of SMPs with density (e.g., polyethylene [PE; 910–930 kg m^–3^] and polypropylene [PP; 830–850 kg m^–3^] lighter than seawater [approximately 1025 kg m^–3^]) decreases exponentially with depth when an equilibrium is accomplished between buoyancy-driven rising and vertical diffusion.^22^ Let us consider a scenario in which the settling of SMPs due to the aforementioned biological processes predominates over horizontal transport, vertical diffusion, and buoyancy-driven rising, which depends on particle size and density. This scenario is plausible, as the settling velocity of suspended organic matter potentially incorporating SMPs is 1–100 m per day in the open ocean,^23^ which would allow SMPs to reach depths of 1000 m within approximately 10 days to 3 years. Therefore, a vertically homogeneous SMP profile is likely to emerge in a steady state, if SMPs have been continuously introduced to the uppermost ocean layer in recent decades. In fact, SMP concentrations at the surface are comparable to those at 5000 m in the Arctic Ocean.^18^ Nonetheless, the actual situations are more complex than expected in a simple settling process. SMP maxima at depths below the euphotic layer have been found at depths greater than 1000 m,^19^ and around 2000 m depth,^20^ suggesting that the transport pathways for these subsurface plastics remain poorly understood.

The objective of the present study is to investigate both vertical and horizontal transport pathways of SMPs in open oceans based on 12-layer seawater sampling and hydrographic surveys from the sea surface to 1000 m in depth in the western North Pacific Ocean. An advantage of the present study over previous SMP studies (sampling in one to six layers) was the 12-layer sampling, which enables comparison of SMP vertical profiles with water mass structures revealed in hydrographic surveys. Lightweight (as described above) and fresh SMPs not yet affected by biological processes can move horizontally in the surface ocean circulation such as the North Pacific subtropical gyre. Meanwhile, SMPs via biological processes in the surface euphotic layer as well as polymers such as polyester and polyamide (also known as nylon) are heavier than seawater and thus potentially sink to the abyssal ocean. The SMPs that have reached near-neutral buoyancy through biological processes likely migrate with the seawater along isopycnal surfaces in subsurface layers. Based on a simple modeling approach in combination with field surveys, this study demonstrates how SMPs spread among surface and subsurface layers of the ocean through a combination of these transport processes.

## 2 Methods

### 2.1 Field Surveys

SMP sampling was conducted at four stations concurrently with hydrographic surveys from November to December 2022 in the western North Pacific Ocean using the training vessel Umitaka-maru, belonging to the Tokyo University of Marine Science and Technology (Figure 1; see Table S2 for sampling dates and positions). Stations 1, 2, and 4 were located in the anticyclonic North Pacific subtropical gyre southeast of the Kuroshio Current, while Station 3 was located in the eastward North Equatorial Countercurrent.^24,25^

**Figure 1:**
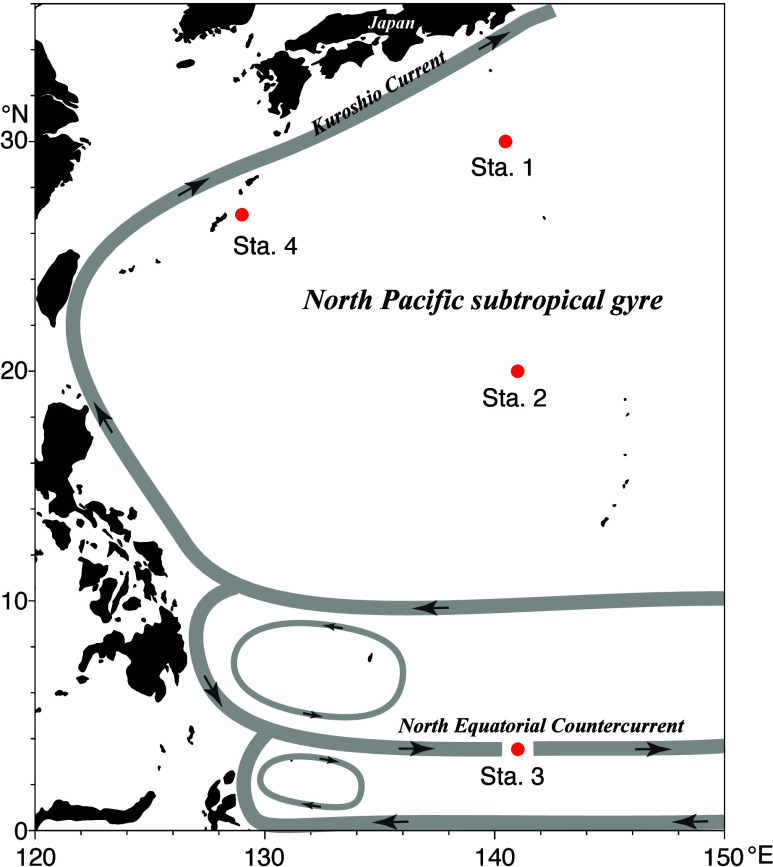
Observation stations (red dots) in the western North Pacific Ocean. Surface ocean circulation and eddies are schematically shown by gray bands.^24,25^

Seawater samples potentially containing both SMPs and natural suspended particles were collected at 12 predetermined depths (0, 10, 20, 30, 50, 100, 150, 200 [260 only for Station 3], 400, 600, 800, and 1000 m) using 8 L polyvinyl chloride (PVC) Niskin bottles installed to a 24-bottle rosette sampler system during each upcast. We collected seawater samples twice at each depth, resulting in a total of 16 L of seawater. The seawater was directly transferred from the Niskin bottles to polycarbonate (PC) containers on the ship’s deck via a silicon tube to minimize air exposure (Section S1 and Figure S1 for the detailed protocol and photographs, respectively). One advantage of direct seawater sampling is the minimization of SMP loss or fragmentation that might occur within the sampling apparatus.

Although, as mentioned above, the multilayer sampling across 12 layers allows for comparison of vertical profiles of SMP concentrations with the water mass structure in the water column, we sampled a relatively small volume of seawater (16 L) at each depth, which is 1–2 orders of magnitude smaller than those of previous studies using WTS-LV pumps (Table S1 for filtered volume in each study).^18–20^ Small-volume sampling using Niskin bottles results in more variable abundance estimates than the actual values: a comparison between a 5 L Niskin bottle and pump-based MP samplings.^26^ To mitigate the uncertainty caused by such small samples, rather than examining the vertical profile of SMPs at each station, we instead assessed profiles synthesized across multiple stations. Seawater temperature and salinity were concurrently observed using a conductivity, temperature, and depth (CTD) sensor (SBE 911plus, USA; no chlorophyll *a* measurements) installed at the bottom of the rosette sampler. The temperature and salinity were recorded once per meter during descending casts.

### 2.2 Shipboard Sample Processing

Suspected SMPs were extracted from seawater samples along with natural suspended particles using plastic-free filtration equipment in a clean booth (Level 7; Kamata Industry, Japan) installed on the ship. First, seawater was transferred from the PC container onto a stainless-steel filter (10 μm, 47 mm diameter) in a filter holder via a silicon tube with an inner diameter of 7.94 mm (Section S2 and Figure S2 for the detailed protocol and photographs, respectively). Thereafter, the filter was rinsed through suction filtering and placed in a perfluoroalkoxy alkane (PFA) container. The inside wall of the stainless-steel filter holder was rinsed with 99.8% ethanol solution (hereinafter, “ethanol solution” except where otherwise stated), which then was suction-filtered onto another stainless-steel filter to extract suspected SMPs potentially remaining in the ethanol solution. This second filter was placed in the same PFA container as the first filter. The containers containing filters were covered with aluminum foil and transported to the laboratory.

### 2.3 Sample Processing in the Laboratory

In the laboratory, two-step digestion at low temperatures^27^ was employed to reduce physical and chemical damage to SMPs. Both stainless-steel filters in the PFA container were soaked in a tall beaker with 30 mL of 10% KOH solution at 40 °C for 72 h, followed by oxidative digestion with 60 mL of 30% H_2_O_2_ + 20 of mL Fe^2+^ 0.05 M at 40 °C or less. Thereafter, the filters were rinsed with Milli-Q water followed by the ethanol solution, and the liquid was collected in a tall beaker. SMPs that potentially remained on the filter surface were transferred to the ethanol solution using an ultrasonic cleaner. Finally, the ethanol solution was transferred to the same tall beaker and filtered through a polytetrafluoroethylene (PTFE) filter for subsequent plastic polymer identification (Supporting Information, Section S3 for the detailed process).

Plastic polymer types were identified using micro-Fourier transform infrared spectroscopy (μFTIR; Thermo Fisher Scientific, Nicolet iN10 MX; see the Supporting Information and Figure S3 for the detailed process and photographs, respectively). The polymer type was identified for each suspected SMP through comparison of its infrared absorption spectrum with those archived in spectrum libraries in accordance with two criteria: hit quality larger than 60% and the presence of all peaks expected for the polymer type.^19^ Polymer types with high production percentages and that have frequently detected in previous SMP surveys (e.g., Shim et al.^28^) were preselected for identification to streamline processing. PP, PE, polypropylene copolymer (PEP), and ethylene-vinyl acetate (EVA) with densities lighter than seawater were targeted. Ethylene propylene rubber (EPDM) was also identified despite having a production percentage much smaller than other polymers, as this polymer type is often misclassified as PE due to its similar infrared absorption spectra (Figure S4). In addition, PS (960–1050 kg m^–3^), which is used for expanded PS foam and frequently found in marine debris,^29^ was included among the polymer types classified in μFTIR analysis. Polyester (also known as poly­(ethylene terephthalate) [PET; 1370 kg m^–3^] and polybutylene terephthalate [PBT; 1310 kg m^–3^]) is unlikely to move a long distance in the oceans due to its high density. Nonetheless, it was included among the polymers identified, as polyester SMPs fibers can reach the upper ocean via atmospheric deposition.^30^ However, nylon, widely used worldwide, was excluded due to the high content in the blank samples, as discussed below (Section 3.1). Moreover, we excluded the plastic polymers listed in Table S3 from the identification process because they were used in the field surveys and subsequent sample processing.

SMP size was defined by the Ferret maximum diameter of each particle visible on the monitor display and was measured using image-processing software provided with an μFTIR instrument. The resolution of SMP sizes was estimated to be approximately >10 μm based on calibration using SMP sizes measured with a stereoscopic microscope (Olympus, Japan, SZX7).

### 2.4 Tests for Contamination, Recovery Rate, and Breakage Rate

In addition to quality assurance and quality control to reduce SMP contamination (Supporting Information, Section S4), we created three blank samples to estimate potential SMP contamination on the ship and/or in the land-based laboratory. First, to evaluate potential contamination from airborne sources in the clean booth installed onboard the ship, two PFA cups filled with Milli-Q water were placed near the filtration equipment during the seawater sample filtration process. Second, 2 L of Milli-Q water stored in cleaned glass bottles was carried from the ship to the land-based laboratory to detect SMPs originating from the onboard Milli-Q water system. Third, to detect contamination from the equipment interior, 5 L of Milli-Q water free of SMPs was processed in triplicate via filtration in the onboard clean booth, two-step digestion, and filtration onto PTFE filters prepared for μFTIR. Conducting a blank test for the interior of the Niskin bottles was considered unnecessary, as the inner walls were likely rinsed by seawater that flowed through the bottles during the 1000 m downcast, prior to sample collection on the upcast. Nevertheless, to eliminate any SMPs potentially adhering to the inner surfaces, all Niskin bottles were thoroughly washed with neutral detergent and subsequently rinsed with tap water, Milli-Q water, and ethanol solution before each deployment (Section S4).

Estimating the percentage of breakage is valuable for reducing the overestimation of particle counts due to unexpected fragmentation during sample processing. Breakage tests were conducted in triplicate using 50 μm spherical high-density PE beads of red for easy visual identification. PE beads were selected for this test due to both the high abundance of PE in ocean plastics^28^ and their vulnerability to breakage compared to other polymers; for example, the tensile strength of high-density PE (PS) is 23–31 (36–52) MPa. In total, 170, 246, and 325 PE beads in each trial were processed through the steps of filtration, two-step digestion, and final filtration on a PTFE filter. Finally, we counted the number of unbroken PE beads after sample processing by using a stereoscopic microscope.

To determine the recovery percentage of SMPs in our protocol, 100 spherical PS beads with a diameter of 100 μm were processed in triplicate through filtration, two-step digestion, and final filtration onto PTFE filters prepared for μFTIR. Red spherical beads were used for the test to distinguish from SMPs potentially contaminated in the processes. To examine size dependency, the recovery percentage was computed by dividing the sum of broken and unbroken 50 μm PE beads used for the evaluation of the breakage percentage by the original number of beads.

Although blank tests and/or careful exclusion of potentially contaminated SMPs during sampling and laboratory processes were conducted in previous studies (Table S1), the breakage and recovery percentages were not evaluated. Therefore, the procedures for estimating these percentages and applying subsequent data corrections (as described in Section 3.1) will be useful for SMP surveys where protocols are not yet well established.

## 3 Results

### 3.1 Contamination, Recovery Percentage, and Unbroken Percentage in Our Protocol

Overestimation of the SMP abundance might occur due to the contamination of SMPs from ambient air and/or equipment. No SMPs were found in the two blank samples installed in the clean booth on the ship. However, in the land-based laboratory, nylon SMPs whose source(s) could not be identified were detected in all three trials (27, 15, and 39 pieces); therefore, we excluded nylon SMPs from subsequent analysis as well as other plastic polymers used in sample processing (Table S3). Excluding the nylon contamination, an average of 5.3 contaminating SMPs consisting of PE (0.67 pieces), PP (2), PEP (0.67), and PET (2) fragments arose during the entire process.

Overestimation (or underestimation) of SMP abundance might occur due to SMPs being broken or lost during processing. Unbroken spherical beads accounted for 86.9% of the averaged across three trials. The recovery percentage of 100 μm spherical beads was 87.8% across three trials, while that of 50 μm spherical beads was 88%, essentially equal to the percentage obtained in the tests using 100 μm beads. In accordance with a fragmentation model,^31^ a formula to convert the observed particle count (*N*) to the corrected particle count (N*) was developed as N* = (*N* × 0.68 – 5.3)/0.88 (Section S5). For reference, corrected values are also shown in the following sections, although the differences between the observed and corrected values were minimal.

### 3.2 Abundance, Sizes, Shapes, and Polymer Types of SMPs

At four stations, 36–230 of SMPs were collected at each depth by 16 L seawater sampling. The particle count averaged over all samples was approximately 110 particles (Table S4). Although approximately 5% (5.3/110) of the entire particle count represented potential contamination, as suggested by the blank tests described above, overestimation due to contamination was considered negligible. The SMP concentrations observed at each depth at four stations ranged from 2250 to 14,375 particles m^–3^, with an average of 6910 particles m^–3^ (Table S4), larger than the values reported in previous studies (Table S1). However, our observations and subsequent sample processing protocols indicated that the true SMP abundance might be only approximately 70% of the observed values (i.e., 4800 particles m^–3^), in accordance with the formula (110 × 0.68 – 5.3)/0.879 = 79 of SMPs. The SMP abundance of the order of 10^3^ to 10^4^ particles m^–3^ was observed in the marginal seas of the western North Pacific Ocean (Table S1),^15,16,21^ where an elevated abundance of MPs has been reported,^32^ attributed to the largest riverine plastic emissions within the North Pacific.^33^ Nonetheless, it is worth noting that the SMP abundance in the eastern North Pacific,^20^ including the Great Pacific Garbage Patch (GPGP),^34^ is an order of magnitude lower than that in the western North Pacific.

The average (or median) size of the SMPs collected in all surveys was 92 (or 56) μm. SMPs <100 μm, which accounted for 79.3% of the total, were present at all depths (Figure S5). If the vertical distribution of SMPs was determined as an equilibrium state between upward motion and vertical diffusion, then SMP sizes would decrease downward due to reduced upward motion. This is because the e-folding depth (= *K*/*W*), where *K* is the vertical diffusivity and *W* is the upward velocity proportional to the square of the particle size, increases as the particle size (and thus the *W*) decreases. However, this was not the case. A size decrease in deeper layers was not clearly observed at all stations (Figure S6). Rather, SMPs observed in 11, 8, and 5 layers at Stations 1, 3, and 4, respectively, were larger than those observed at 0 m depth with statistically significant differences by the tests described in Section S6.

In terms of shape, a majority of SMP were fragments, accounting for 89–95% of SMPs collected at the four stations (Figure S7). However, as the size of SMPs decreases, visually distinguishing hard-plastic fragments from fibrous pieces on the monitor display of the μFTIR become difficult. Most fibers observed in the present study were concentrated in the size range above 300 μm with an average (or median) size of 445 (or 339) μm (data not shown in figures). Therefore, we do not distinguish between fibers and fragments in the following sections.

Polymer types that are less dense than seawater (e.g., PP, PE, and EPDM suspected to PE) accounted for approximately 70% of the total, averaged over the entire water column (Figure S8). This suggests that these lightweight SMPs, which would normally be trapped in the surface layer, become neutral or heavier than ambient seawater via biological processes. Nonetheless, polyester SMPs with a density heavier than seawater accounted for the remaining 30% and were widespread throughout the water column at offshore stations.

### 3.3 Vertical Distribution of SMPs

An advantage of this study is that the 12-layer seawater sampling allowed us to compare the vertical profiles of SMP concentrations and hydrographic properties (Figure 2). The potential temperature and density curves demonstrated a mixed layer developed from the sea surface to depths of 50–200 m at four stations during boreal winter. Subsurface peaks of approximately 10,000 pieces m^–3^ were revealed in SMP concentrations at depths of 1000 m at Stations 1 and 2, 400 and 800 m at Station 3, and 800 m at Station 4. Except in the equatorial Pacific Ocean (Station 3), these high concentrations appeared below the salinity minimum, representing the North Pacific Intermediate Water (NPIW), which is a water mass characterized by a subsurface salinity minimum that is widely distributed in the North Pacific subtropical gyre.^35^ The lower-salinity water mass comes from the subarctic area where precipitation exceeds evaporation and is subducted between isopycnal surfaces with potential densities of 26.6 and 27.0σ_θ_ (around 600 m depth in the areas of the present study) in the North Pacific Ocean.^35,36^ NPIW is the densest and deepest water mass ventilated in the North Pacific Ocean.^35^

**Figure 2:**
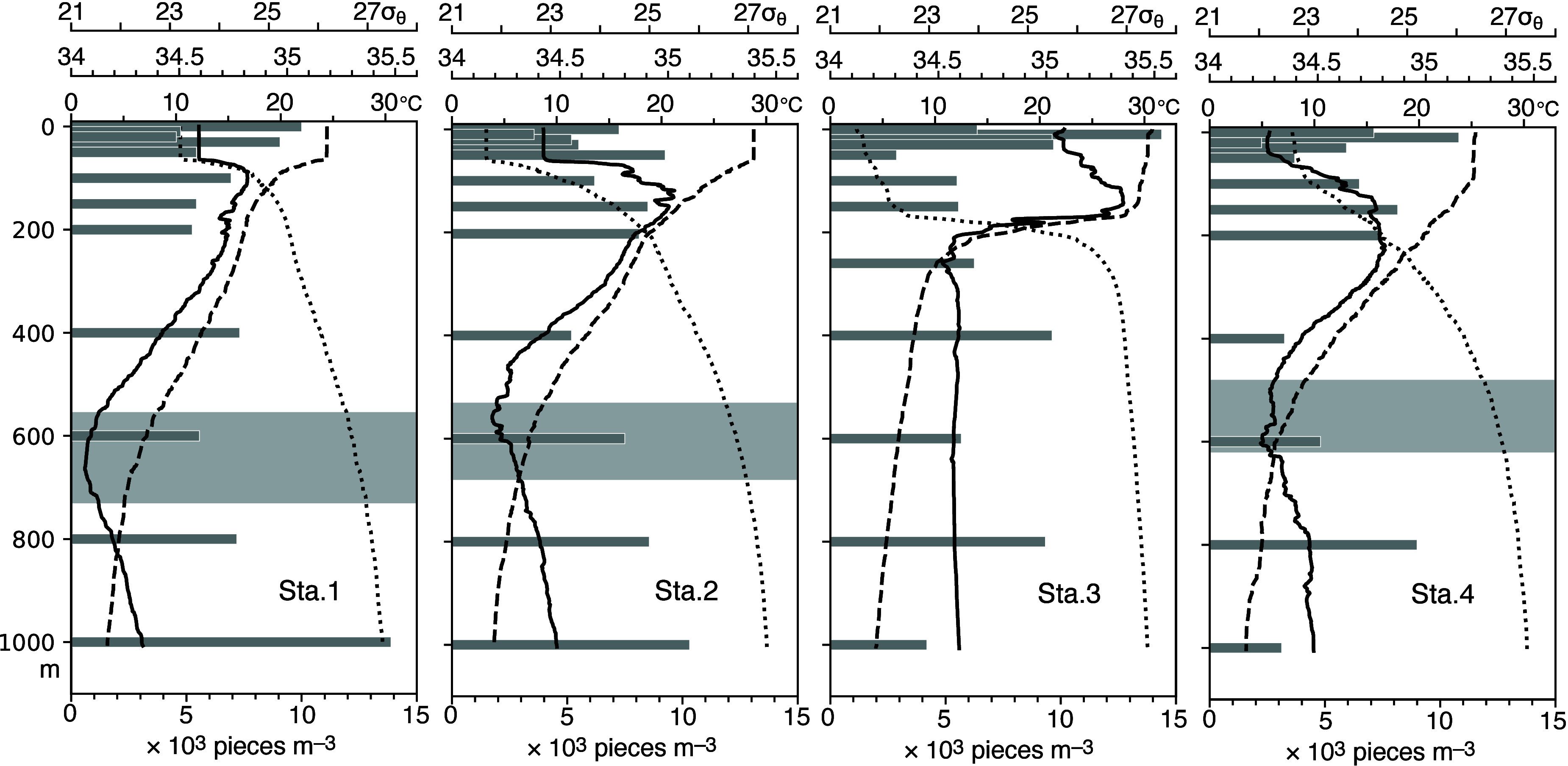
Vertical distribution of SMP concentrations (bars; bottom abscissa) superimposed on potential temperature (broken curve), salinity (solid curve), and potential density (dotted curve) at four stations. The upper, middle, and lower abscissae at the top of each panel represent the potential density in σ_θ_, salinity, and potential temperature, respectively. Stippling denotes the isopycnal layers (26.6–27σ_θ_) along which NPIW is subducted.^35^

## 4 Discussion

### 4.1 Vertical Distribution of SMPs and Its Possible Drivers

Polyester SMPs were excluded from the subsequent analyses despite constituting a major fraction of all polymer types (Figure S8) because their relatively high density suggests that they are unlikely to travel long distances in the ocean. According to Stokes’ law for spherical bodies, terminal velocity can be estimated using a median SMP size of 56 μm, seawater density (1025 kg m^–3^), polyester density (1380 kg m^–3^), gravitational acceleration, and seawater viscosity (1.025 × 10^–3^ kg m^–1^ s^–1^). Substituting these values into δ, ρ_s_, ρ′, *g*, and η, respectively, in δ^2^(ρ_s_ – ρ)*g*/18η yields a terminal velocity of *O*(10^–3^) m s^–1^, allowing particles to sink from the sea surface to 1000 m depth in approximately 10 days. Thus, polyester SMPs likely follow different pathways than other SMPs (e.g., atmospheric deposition followed by rapid settling) and are unlikely to be transported >100 km by ocean currents (typically 0.1 m s^–^^1^) from land sources.

The similarity between the vertical profiles of SMPs (except polyester) and salinity is notable, suggesting that the SMP pathway is related to water masses such as NPIW in the subtropical gyre (left panel in Figure 3). Station 3 was located in the equatorial Pacific Ocean, where ocean circulation—and therefore the transport pathways of SMPs—differs from that in the subtropical gyre. Therefore, only the profiles from the three stations located within the subtropical gyre (Stations 1, 2, and 4) are superimposed in the left panel of Figure 3 (>300 particles per 16 × 3 = 48 L seawater at each depth). Isopycnal coordinates are used for the ordinate, following conventional oceanography, as the depths of isopycnal surfaces differ among stations. Overall, lightweight SMPs were more abundant in the 23–25σ_θ_ isopycnal layers than above the 22σ_θ_ isopycnal layer despite floating SMPs and atmospheric deposition being associated with the uppermost layer. SMP concentrations increased from the 23 to 24σ_θ_ isopycnal layer, suggesting that the vertical profiles of SMPs were not determined simply by downward settling from a surface layer containing abundant SMPs. The SMP concentration rapidly decreased from the 25 to 27σ_θ_ isopycnal layers. In the 22–27σ_θ_ isopycnal layers where observations above NPIW were conducted at three stations, the correlation coefficient between the SMP concentration and salinity—both influenced by along-isopycnal subduction—was 0.50 and statistically significant, as suggested by a *t* test with a 95% confidence level (Figure S9).

**Figure 3:**
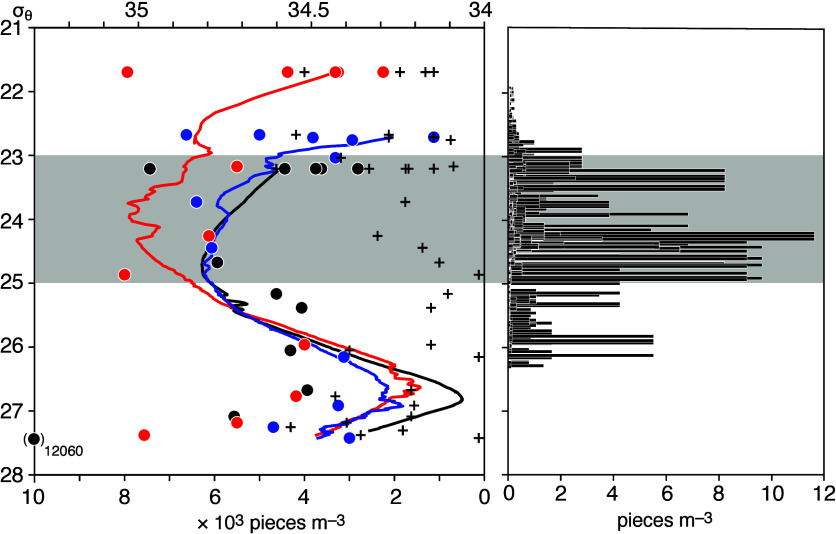
Comparison between SMP concentrations (particle count per unit seawater volume) in the subsurface layer (left) and MP concentrations at the sea surface in the same isopycnal layers (right). SMP concentrations excluding polyester (dots) are shown alongside salinity distributions (curves) at Stations 1 (black), 2 (red), and 4 (blue). The crosses denote concentrations of the polyester SMPs. MP concentrations (downloaded from the Atlas of Ocean Microplastics [AOMI] database; https://aomi.env.go.jp; >4000 data in the North Pacific) in the same isopycnal layers outcropped at the sea surface (Figure S10) are indicated by bars. Stippling denotes isopycnal layers between 23 and 25σ_θ_, as shown in Figure S10.

Meanwhile, the correlation coefficient between the polyester SMP concentration (left panel in Figure 3) and salinity in the same isopycnal layers was 0.20, an insignificant value suggested by a *t* test with a 95% confidence level. Of particular interest is that polyester SMP concentrations were not vertically homogeneous, contrary to our expectation for SMPs being significantly denser than seawater. In the present study, we did not investigate the reasons behind the high concentrations observed in both the upper (<23σ_θ_) and lower (>27σ_θ_) isopycnal layers. However, further exploration is necessary to better understand the sources and fate of heavy SMPs in the ocean.

Let us consider SMP behavior in subsurface layers in the framework of the ventilated thermocline theory^37^ of classical ocean dynamics. Lightweight SMPs originally floating at the sea surface have opportunities to encounter isopycnal layers that outcrop at the sea surface. Simultaneously, floating SMPs are subjected to biological processes that increase their density. Since the observations in this study were conducted in oligotrophic open ocean regions, the biological processes are considered to have occurred in other nutrient-rich regions. As a result, along-isopycnal subduction from the sea surface to the ocean interior emerges as a plausible transport pathway for SMPs exhibiting near-neutral buoyancy and negligible settling velocity due to their density being close to that of seawater. Meanwhile, subsurface SMPs are likely to be independent of isopycnal layers if they exhibit marked settling across isopycnal layers (see the concentrations of polyester SMPs in the left panel). The 23–25σ_θ_ isopycnal surfaces with abundant SMPs in the subsurface layer (left panel in Figure 3) outcrop at the sea surface during boreal winters, and floating MPs are also abundant due to surface convergence in the North Pacific subtropical gyre including the GPGP northeast of the Hawaiian Islands (Figure S10).^34,38,39^ Under the assumption that fresh SMPs not yet affected by biological processes are also abundant in the surface convergence zone, the surface SMPs of which density increases gradually via biological processes are suggested to migrate below the sea surface through along-isopycnal subduction. MP concentrations at the sea surface between the 23 and 25σ_θ_ contour curves are larger than those observed in surrounding isopycnal layers (right panel in Figure 3) in a fashion similar to that of the SMP vertical profile in isopycnal coordinates (left panel in Figure 3).

Notably, elevated SMP concentrations were frequently revealed below the NPIW with a salinity minimum around 26.6–27.0σ_θ_ isopycnal layers (left panel of Figure 3), along which the lower-salinity water mass formed in the subarctic region spreads across the North Pacific subtropical gyre.^35,36^ Meanwhile, concentrations higher than 5 × 10^3^ pieces m^–3^, which were observed below the NPIW, were not detected in the 25–27σ_θ_ isopycnal layers. Importantly, isopycnal layers below the NPIW never outcrop at the sea surface throughout the year^35^ and thus have no chance to encounter SMPs floating at the sea surface. SMPs below the NPIW follow no pathway other than settling across the isopycnal surfaces from the upper layers.

The SMP abundance at Station 3 in the equatorial Pacific Ocean also shows a vertically inhomogeneous distribution, which might be related to water masses, similar to what is observed in the subtropical gyre. However, this paper does not explore the mechanisms behind the vertical inhomogeneity, as further surveys at multiple stations in the equatorial Pacific are required.

### 4.2 Subsurface Pathways of SMPs Undergoing Weak and Strong Settling

#### 4.2.1 Model Description

A simple model representing the North Pacific subtropical gyre is useful for demonstrating how SMPs spread in subsurface layers along the isopycnal surfaces and revealing when the SMP subduction occurs under actual conditions. Let us consider a two-dimensional domain surrounded by sidewalls at both ends (Figure 4). The ocean contains multiple layers (from *k* = 1 at the surface to *k*_m_ increasing downward), all of which outcrop at the sea surface. Below the *k*_m_th layer, we imposed an isolated bottom layer (*k* = *k*_m_ + 1) that does not outcrop at the sea surface, representing isopycnal layers below the NPIW. The SMP concentrations in the *k*th layers (*C*_*k*_) were computed as described below.

**Figure 4:**
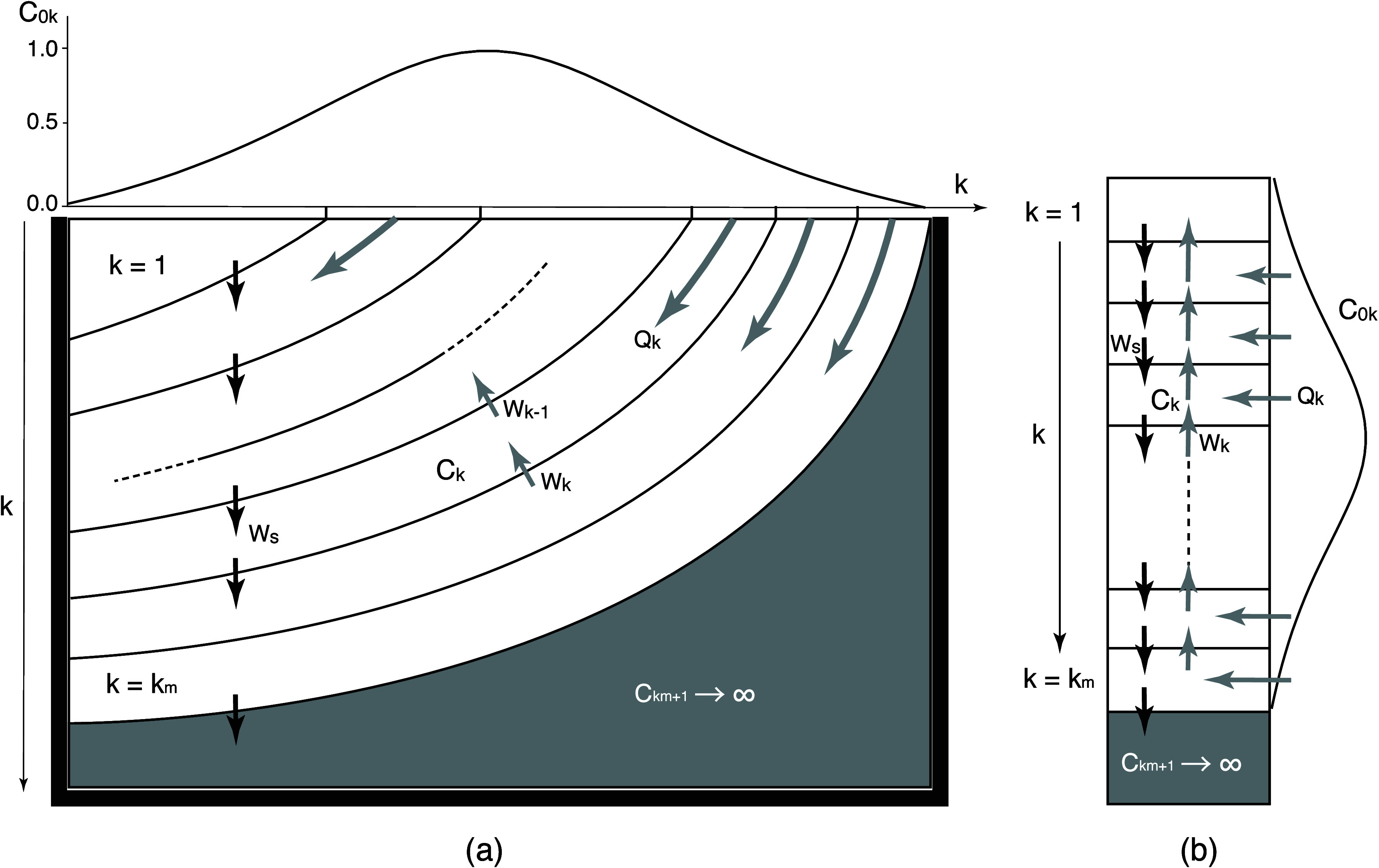
Model used to reproduce the isopycnal subduction of SMPs from the sea surface to the subsurface layers. Note that the two-dimensional processes depicted in panel (a) are essentially reduced to one-dimensional processes in panel (b) in the context of the present model experiment.

As illustrated in Figures S5 and S8, both the size distribution and plastic polymer composition are assumed to be invariant over the model domain for simplicity. Thus, in the steady state, the mass conservation of SMPs in the *k*th layer is expressed as follows:

$$Q_{k}C_{0k}-W_{k-1}C_{k}+W_{k}C_{k+1}+W_{s}C_{k-1}-W_{s}C_{k}=0$$ 1

where the number of isopycnal layers *k* increases toward the north (or deeper) in the horizontal (or vertical) direction. *Q*_*k*_ and *C*_0*k*_ denote the isopycnal transport of subducted seawater and surface SMP concentration in the *k*th isopycnal layer, respectively, and *W*_*k*_ indicates the diapycnal transport (not necessarily vertical) between the *k* and *k* + 1 isopycnal layers. The isopycnal transport values (*Q*_*k*_) in all layers are directed toward the south in the Sverdrup equilibrium. The continuity equation of the present model expresses the relationship between *Q*_*k*_ and *W*_*k*_ as follows:

$$W_{k}=\sum_{k^{’}=k+1}^{k_{m}}Q_{k^{’}}$$ 2

Note that the diapycnal transport between the *k*_m_th (= 50 in the present model) and bottom layers is nonexistent due to fluid continuity. For simplicity, the settling transport (*W*_s_) across isopycnal layers was constant over the entire model domain. Substituting eq 2 into eq 1 yields

$$C_{k}=\alpha C_{0k}+\beta C_{k-1}+\gamma C_{k+1}$$ 3

where α, β, and γ represent *Q*_*k*_/*F*, *W*_s_/*F*, and 1−α–β, respectively, while *F* denotes ( $\overset{k_{m}}{\underset{k^{’}=k}{\sum }}Q_{k^{’}}$ ) + *W*_s_. Assuming oceanic isostacy in the bottom layer,^37,40^ we imposed a simple linear profile on subducted isopycnal transport (i.e., *Q*_*k*_ = *Q*_max_(1 – *k*/*k*_m_)). The choice of *Q*_max_ could be arbitrary because it was excluded in eq 3 when we set *W*_s_ proportional to *W*_*k*_ (hence, *Q*_*k*_ in eq 2), as described below. Given a distribution symmetrical to the surface SMP concentration (*C*_0*k*_ = 1/{1 + 10­(*k*/*k*_m_ – 0.5)^2^}), we obtain *C*_*k*_ in eq 3 through iteration for *k* > 1 (*C*_1_ was fixed to *C*_01_). The distribution of *C*_0*k*_ is also arbitrary, representing the dense SMP presentation at midlatitudes (Figure 3 and Figure S10). The boundary condition, *C*_0*k*_, was assumed to be based on a combination of microplastic emissions, horizontal transport in the surface layer (i.e., *k* = 1), and upward transport of microplastics from the immediately underlying layer (i.e., *k* = 2), although these processes were not explicitly represented in the model.

The objective of the model computation is determining how the vertical SMP distribution (*C*_*k*_; left panel in Figure 3) is generated via the subduction of surface SMPs (*C*_0*k*_; suggested by MPs in the right panel of Figure 3). Actual surface SMP concentrations could not be reproduced, as neither lightweight polymer plastics nor atmospheric deposition were included in the present model. The concentration in the bottom layer is infinitely large (*C*_*k*m+1_ → ∞), and the bottom layer can be considered as a dead end for SMPs settling from upper layers. In fact, elevated SMP concentrations were frequently revealed below the NPIW (Figure 3), which were considered to have been carried from the upper layer, either near the observation stations or elsewhere. An infinitely dense concentration never appears in reality due to sedimentation into the ocean floor,^41,42^ which is excluded from the present model.

In this study, we examined three experimental cases with different settling transport values (*W*_s_). First, SMPs reach a neutral density via biological processes and therefore the settling transport was set to be around zero (*W*_s_ = 0 ± 0.1 $\overset{-}{W_{k}}$ , where ±0.1 $\overset{-}{W_{k}}$ suggests a weak upward/downward motion to examine the model sensitivity). Second, settling transport is comparable to the typical diapycnal transport generated in ambient water, *W*_s_ = $\overset{-}{W_{k}}$ , using the diapycnal transport averaged across all layers. Third, the settling transport affected by biological processes is much greater than diapycnal transport in ambient water, *W*_s_ = 10 × $\overset{-}{W_{k}}$ , which is likely for typical settling velocities (1–100 m/day) of particulate organic matter in the open oceans.^23^

#### 4.2.2 Isopycnal Transport of SMPs Subducted from the Sea Surface

The vertical distribution of concentrations (*C*_*k*_; left panel in Figure 5) converted from those at the sea surface (*C*_0*k*_; right panel in Figure 5) via along-isopycnal subduction was sensitive to the selection of the settling transport value (*W*_s_). In the case of near-neutral buoyancy (*W*_s_ = 0 ± 0.1 $\overset{-}{W_{k}}$ , the symmetry of the concentrations at the sea surface was generally preserved in the vertical profile, although upward transport (*W*_*k*_) over the model domain distorted the symmetry by moving the peak upward. As a result, in a fashion similar to the observed concentrations (left panel in Figure 3), the modeled concentrations of neutrally buoyant SMPs increased from the top layer to the subsurface peak and decreased rapidly downward below the peak (left panel in Figure 5). The model suggests that the SMP concentration reaches a minimum (*C*_*k*m_) just above the isolated bottom layer (*k*_m_+ 1), resembling the high SMP concentrations revealed below the NPIW in the real ocean. The rapid decrease below the peak became unclear in the experiment, with settling transport comparable to the upward transport in ambient water (*W*_s_ = $\overset{-}{W_{k}}$ ). When the settling transport was increased 10-fold (*W*_s_ = 10 × $\overset{-}{W_{k}}$ ), concentrations become almost uniform vertically. The concentration peak appeared only in the model with weak settling transport (*W*_s_ = 0 ± 0.1 $\overset{-}{W_{k}}$ ; Figure 5), suggesting that the elevated concentrations observed in the 23–25σ_θ_ isopycnal layers above the NPIW (left panel of Figure 3) were attributable to near-neutrally buoyant SMPs, whose vertical transport was less than 10% of that in the ambient water.

**Figure 5:**
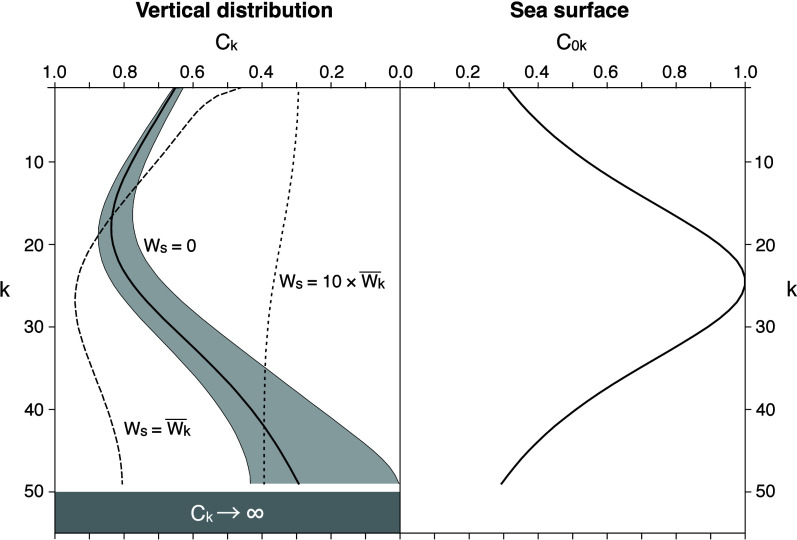
Model results for comparison with Figure 3. Concentrations normalized to the maximum value at the sea surface (right) were converted to vertical profiles with different settling transport values (left). Stippling indicates the range of *C*_*k*_ obtained by varying *W*_s_ by ±0.1 $\overset{®}{W_{k}}$ .

Finally, we propose two settling pathways for SMPs floating at the sea surface (Figure 6). Buoyant SMPs suspended in the surface layer prior to undergoing biological processes are likely to accumulate in convergence zones due to surface ocean currents (Figure S10). While floating at the sea surface, their density gradually increases within the upper euphotic layer as a result of biological processes, including biofouling, absorption into detritus, and absorption into diatom aggregates. The density of SMPs that change in the euphotic layer depends on the progression of biological processes. SMPs that become considerably heavier than seawater undergo strong settling (first pathway; see the Supporting Information, Section S13 for the computation of settling velocities of biofouled SMPs). These SMPs accumulate in isopycnal layers below the NPIW, which never outcrop at the sea surface throughout the year. However, the lower SMP abundance in the eastern North Pacific, including GPGP (Table S1), suggests different pathways other than settling, despite the lack of standardization in sampling equipment and filtration volumes. Others that reach near-neutral buoyancy with respect to seawater follow the second pathway. SMPs that undergo weak settling are subducted into the ocean interior from isopycnal layers that outcrop at the sea surface. Therefore, the global inventory of near-neutral SMPs with weak settling is expected to be large in subsurface layers.

**Figure 6:**
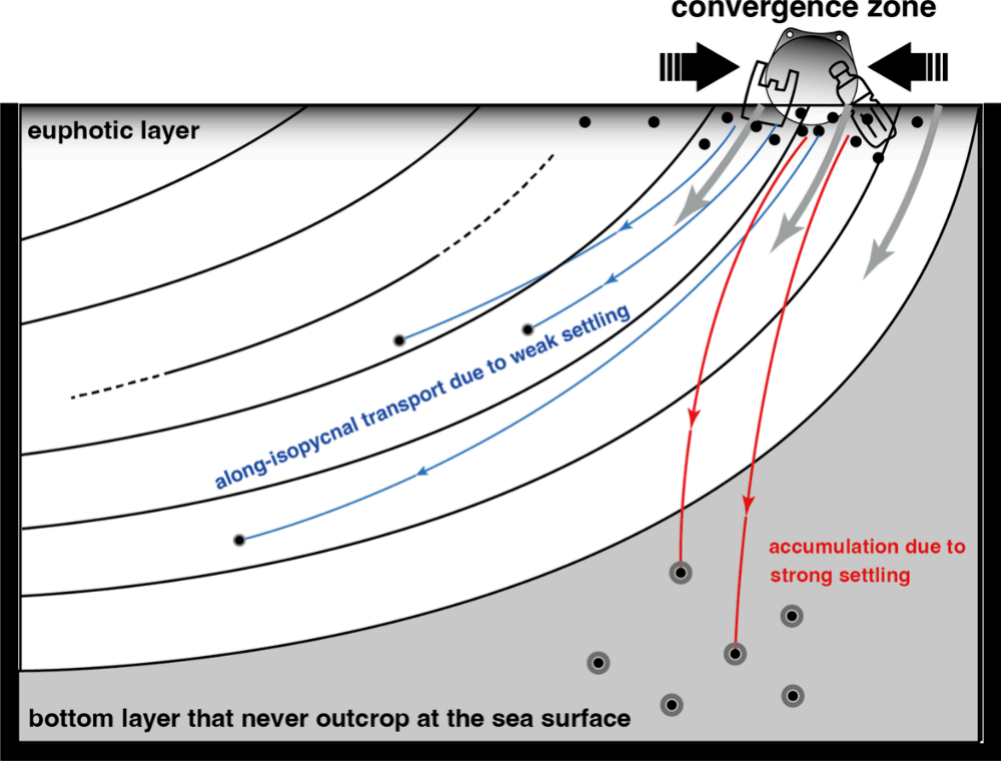
Schematic representation of two pathways followed by SMPs (particles) from the surface convergence zone after biofouling (particles surrounded by gray rings) in the euphotic layer. Weak settling (blue arrows) and strong settling (red arrows) carry the SMPs to subsurface and never-outcropped bottom layers, respectively.

Our results suggest that the pathways and fates of SMPs (and hence of ocean plastics generally) are sensitive to settling speed, which depends on the time that SMPs are exposed to biological processes in the uppermost layers. In our simple modeling approach, only three constant settling transports (*W*_s_ in Figure 5) were investigated for simplicity. In reality, however, speed varies spatially, causing SMPs in waters with high biological activity to become heavy more rapidly than in oligotrophic waters. Furthermore, settling speed varies temporally, both because organic matter attached to the surface of settling SMPs decomposes below the eutrophic layer and because lightweight SMPs such as buoyant PP and PE particles move upward by recovering rise velocities.^43,44^ Descending and ascending motions can occur repeatedly, and such a yo-yo motion potentially makes the fate and pathways of SMPs more complex than those demonstrated in the present study.

## Supplementary Material


