# Supplementary material for: Settling and Along-Isopycnal Subduction of Small Microplastics Into Subsurface Layers of the Western North Pacific Ocean
Source: Environ Sci Technol. 2025 Sep 18;59(38):20716–25. doi: 10.1021/acs.est.5c08983 (PMC12490013; doi:10.1021/acs.est.5c08983)
Supplement: Supplementary file 1 [file es5c08983_si_001.pdf]

Supporting Information

for

"Settling and along-isopycnal subduction of small microplastics into subsurface layers of the western North Pacific Ocean"

Mao KURODA[a], Atsuhiko ISOBE[a*], Keiichi UCHIDA[b], Ryuichi HAGITA[b], Satoru HAMADA[b]

[1] Research Institute for Applied Mechanics, Kyushu University

[2] Tokyo University of Marine Science and Technology

*Corresponding author
E-mail: aisobe@riam.kyushu-u.ac.jp

Outline
The supplementary Information contains 18 pages, which includes 11 figures and 4 tables.

S1. Field Surveys

After we transferred sea water in Niskin bottles to each PC container (Fig. S1), the container was immediately covered with a rubber plug to avoid decontamination from airborne S-MPs. The interior of Niskin bottles was thereafter washed twice using Milli-Q (100 ml) and 60% ethanol (75 ml), respectively, to recover S-MPs remained in the bottles. A relatively low concentration of 60% ethanol solution was used to prevent ignition on the deck. The Milli-Q water and ethanol were transferred to the same PC container.

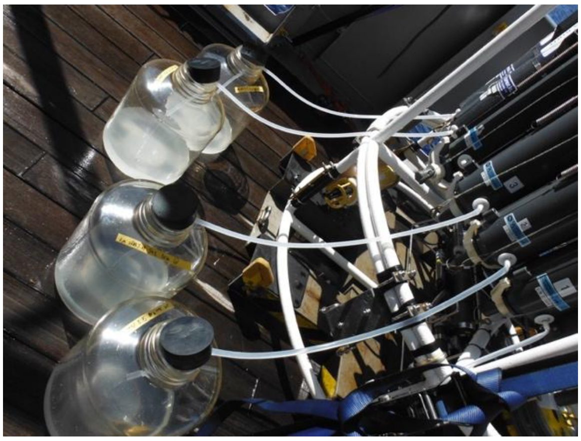

Fig. S1 Seawater samples transferred from Niskin bottles to PC containers

S2. Sample processing on the ship

The method to transfer the sea water from the PC containers to stainless-steel filters was a combination of vacuum pumping (Merck, German, EZSTREAM1) and siphon action to maintain a slow vacuum speed to avoid breaking the potentially weathered S-MPs (Fig. S2, left). To keep the vacuum inside the equipment, the silicon tubes were fixed to the filtration equipment via a custom-made PVC-polytetrafluoroethylene (PTFE) lid (Fig. S2, right). When the seawater transfer was completed, the inside of the silicon tube and PC container, the outside of the silicon tubes in contact with the water sample, and the rubber plug were rinsed with 99.8% ethanol solution to collect any S-MPs that might remain attached to the surface. Then, the accumulated ethanol was suction-filtered on the same stainless-steel filter (mesh size 10 μm, 47-mm diameter) to extract any additional S-MPs. It was found that lipids in seawater can lead to mistakes in polymer identification by spectrometry, as the fatty acids and hydrocarbon chain could be misidentified as polyethylene-type polymers[1]. To avoid this, the stainless-steel filter in the holder was soaked for one minute in ethanol heated to a temperature of 60 °C by a hot plate stirrer. The combination of temperature and time was determined

through trial and error to avoid the destruction of plastic fragments and to remove the substances efficiently.

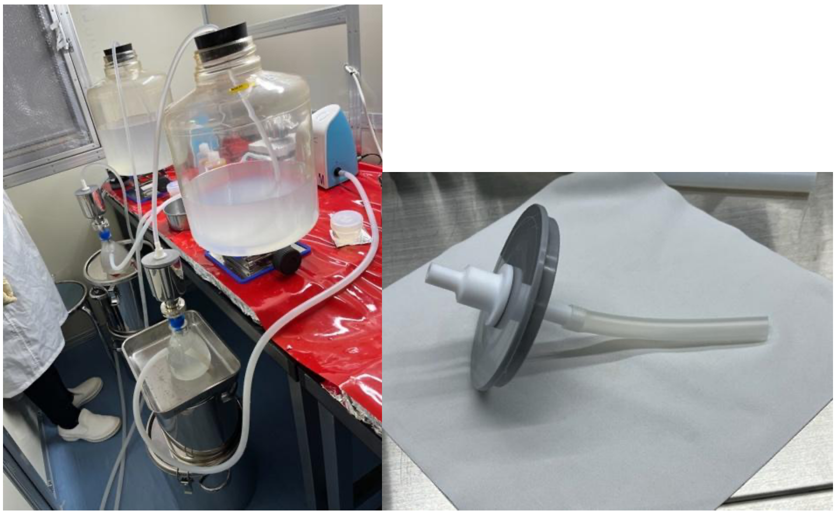

Fig. S2 Filtration by siphon action (left) and custom-made PVC-PTFE lid attached at the top of the stainless-steel filter holder (right).

S3. Sample processing in the laboratory

The stainless-steel filters after the two-step digestion were rinsed first with Milli-Q water and then ethanol solution, and the liquid was collected in a tall beaker. The S-MPs that potentially remained on the filter surface were moved to the ethanol solution using an ultrasonic cleaner. The rinsed stainless-steel filters were put in a PFA container with 30-mL ethanol solution. Then, the capped PFA container was put into an ultrasonic cleaner for one minute to separate all the S-MPs that potentially remained attached to the filter surface. The ethanol solution with the suspected S-MPs was then transferred to the tall beaker and stored for the following steps.

Polymer types, particle count, and sizes of S-MPs were investigated using μFTIR scanning images (obtained by FPA mode, transmission mode, 715 – 4000 cm^{-1} range, 8 cm^{-1} resolution, 16 accumulated scans) in the 8 mm × 8 mm filter area. In general, due to the large amount of data generated using Focal Plane Array (FPA) analysis, only a small area (8 mm × 8 mm) can be scanned each time. For this, it is better to concentrate the S-MPs within a small area in the center of the filter. Filtration was made with a glass filtration unit of 25 mm and PTFE filters of 47 mm diameter. To avoid S-MPs being trapped and lost at the gap between the filter and the filter holder, a custom-made 'support screen' was placed under the PTFE filter used for the suction filtration, leading all the S-MPs to the central area (Fig. S3, upper left). After the filtration, the PTFE filter was fixed to a custom-made metal frame (Fig. S3, upper right) with a rubber ring (Fig.

S3, lower left) and dried in a glass petri dish on the clean bench. Finally, the PTFE filter was set on the micro FTIR sample stage to identify the plastic polymer types of the suspected S-MPs (Fig. S3, lower right).

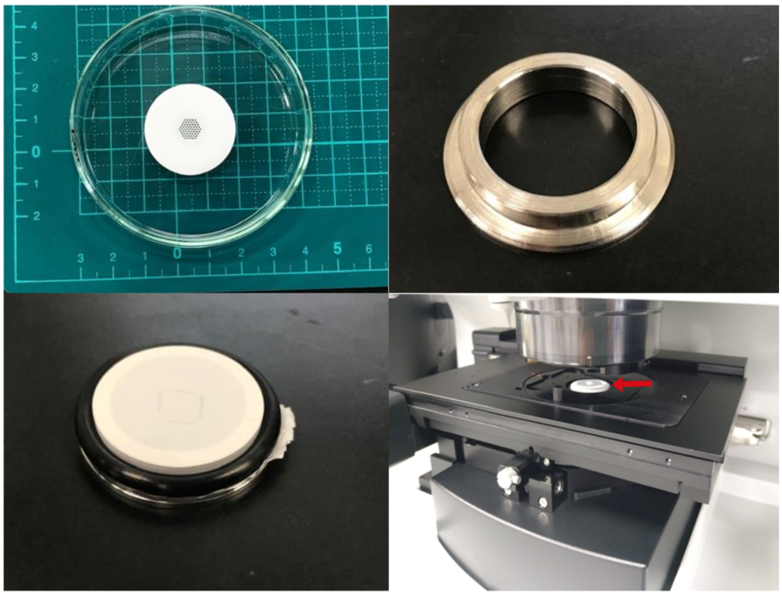

Fig. S3 Preparation of a micro FTIR sample. The support screen (upper left) was set in a metal frame (upper right) beneath the PTFE membrane filter fixed by a rubber ring (lower left) for micro FTIR measurements (lower right, red arrow).

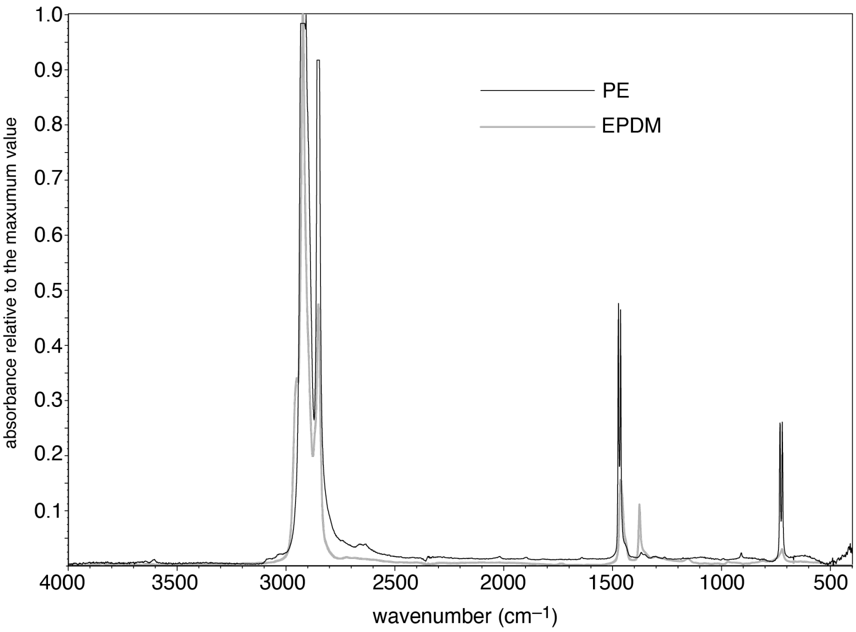

Fig. S4 Examples of PE and EPDM wavenumber spectra measured using μFTIR

S4. Quality assurance and quality control

When measuring SMPs in nature, removing contamination with airborne SMPs that occurs during field surveys and subsequent sample processing is of primary importance. The Niskin bottles were washed with neutral detergent, followed by rinsing with tap water, Milli-Q water, and ethanol solution before each cast. To prevent damage to the bottle interior caused by sponges, the bottles were cleaned by hand (the cleaner wore rubber gloves). In the shipboard clean booth, observers wore clean shoes, clean clothing, and hats during the entire processing time. Both the clean booth and workspaces outside the clean booth were wiped with ethanol solution prior to sample processing. In the laboratory, three high-efficiency particular air filter units were installed to continuously remove airborne dust. Sample processing was conducted on a clean bench with an electrostatic eliminator placed to avoid external dust attachment. SMP contamination from the experimental equipment and reagents was also carefully avoided. Equipment made from silicon resin, fluorine resin, and non-plastic materials such as glass and metals was used in both the shipboard and land-based laboratories with the exceptions listed in Table S3. All filtration equipment was cleaned before use, dried in a drying machine, wrapped in aluminum foil, and stored on stainless steel shelves. Stainless steel equipment was washed with neutral detergent, rinsed with tap water to remove detergent, and then rinsed with Milli-Q water. Glass equipment was neutralized by soaking the glass surface in 0.5% HCl for 1 hour after ultrasonic cleaning with alkaline detergent, followed by rinsing with 0.5% HCl in tap water, and further rinsing with Milli-Q water. Other equipment was first washed with tap water, followed by ultrasonic cleaning for 30 minutes, and then rinsing with tap water to remove cleaning solution, ending with the final rinse with Milli-Q water. Milli-Q water was used to prepare reagents. All reagents used (10% KOH, 30% H_2O_2, 0.05M Fe^{2+}, and ethanol solution) were filtered through a 1 μm PTFE filter to remove potential SMPs and thereafter transferred to a rinsed glass reagent bottle covered with a PTFE lid. This filtration process was repeated until the reagents were free of SMPs, which was confirmed via μFTIR analysis.

S5. Correction of Breakage, Contamination, and Loss in the Sample Processing

In the present study, we attempted to reduce the overestimation due to S-MPs broken in the laboratory processing under the assumption that the fragmentation of MPs occurs according to simple fractal geometries.[2,3] Let us consider an original cube, which can be fragmented to eight equally sized cubes with a probability of P ($0 < P < 1$).

Following Kaandrop et al. (2021) (see their Fig. 2),[3] we assumed that a certain fraction of the original cube splits off in a single fragmentation event with the probability of P, and that these fragmented cubes can each be recursively split again in nature. However, in the laboratory processes with a relatively insufficient time for recursively splitting as in nature, it is assumed that each S-MP splits only once in order from largest to smaller. The selective fragmentation of larger S-MPs seems to be reasonable because the production of smaller pieces requires larger energy, so the probability of the process occurrence becomes lower.[4]

To formulate the correction for breaking S-MPs, let us consider that N particles of S-MPs were observed in a seawater sample, and that n particles out of N particles were broken in the processing. A number of eight-particle groups are fragmented from n original SMPs, corresponding to $8nP$, so that the original number of SMPs before fragmentation (N^*) would be computed as follows:

$N^* = N - 8nP.$ (S1)

Substituting Eq. (S1) into the breakage percentage ($P_b = n/N^*$) obtained from the breakage test (section 2.7) yields

$n = \frac{P_b}{1+8PP_b} N.$ (S2)

Using Eqs. (S1) and (S2), we obtain

$N^* = \left(1 - \frac{8PP_b}{1+8PP_b}\right) N \equiv \gamma N.$ (S3)

Substituting the breakage percentage (100 – 86.9 = 13.1% in section 3.1; $P_b = 0.131$) and the probability P (= 0.45) for PP[3], which was one of prevailing polymer types (Fig. S8), gives a reduction coefficient (γ) of 0.68 in the present study.

Both contamination and loss could be simply corrected using the test results. The overestimation owing to the contamination of S-MPs in the course of the laboratory processing was corrected by subtracting 5.3 particles from the observed particle count of S-MPs irrespective of polymer types. Likewise, the underestimation owing to the loss of S-MPs in the laboratory processes was corrected by multiplying the reciprocal of the recovery percentage [(0.88+0.878)/2 = 0.88]. In our protocol for sampling and sample processing, we can convert the observed particle number (N) to the number of S-MPs drifting in oceans (N^*) as follows:

$N^* = (N \times 0.68 - 5.3)/0.88.$ (S4)

S6. Statistical analysis of the difference in SMP size among depths

If the vertical distribution of SMPs is determined by an equilibrium state between upward motion and vertical diffusion, which is likely the simplest transport mechanism, SMP sizes should be smaller as they go downward due to the low rise velocities of less-buoyant SMPs. Thus, quantitatively comparing SMP sizes across depths is of particular importance. To examine differences in SMP sizes at the sea surface and other depths, a combination of the non-parametric Kruskal–Wallis test and Shirley–Williams' multiple comparison test was employed using BellCurve software for Excel (BellCurve BU, Tokyo, Japan) because SMP sizes at each depth did not follow a normal distribution, as indicated by a Kolmogorov–Smirnov test. Significant differences were assessed at the 99% confidence level for the Kruskal–Wallis test, while significant differences based on the Shirley–Williams' multiple comparison test were assessed at the 95% confidence level, which is widely used for this test.

S7. SMP concentrations by size

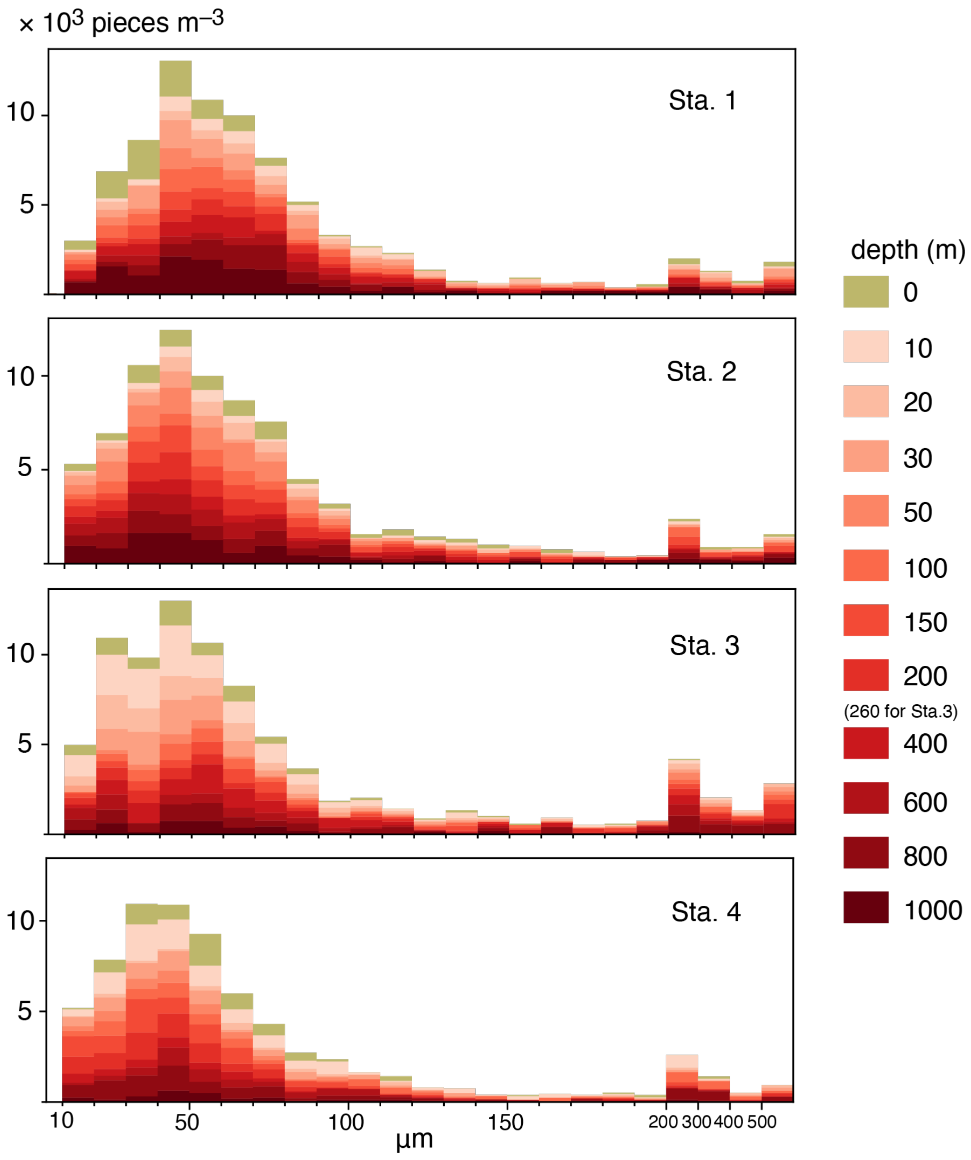

Fig. S5. SMP concentrations by size at four stations. Sampling depths are indicated by the color scale at the right. Note that the sampling depth at Sta. 3 was 260 m, not 200 m.

S8. Sampling depth versus SMP size

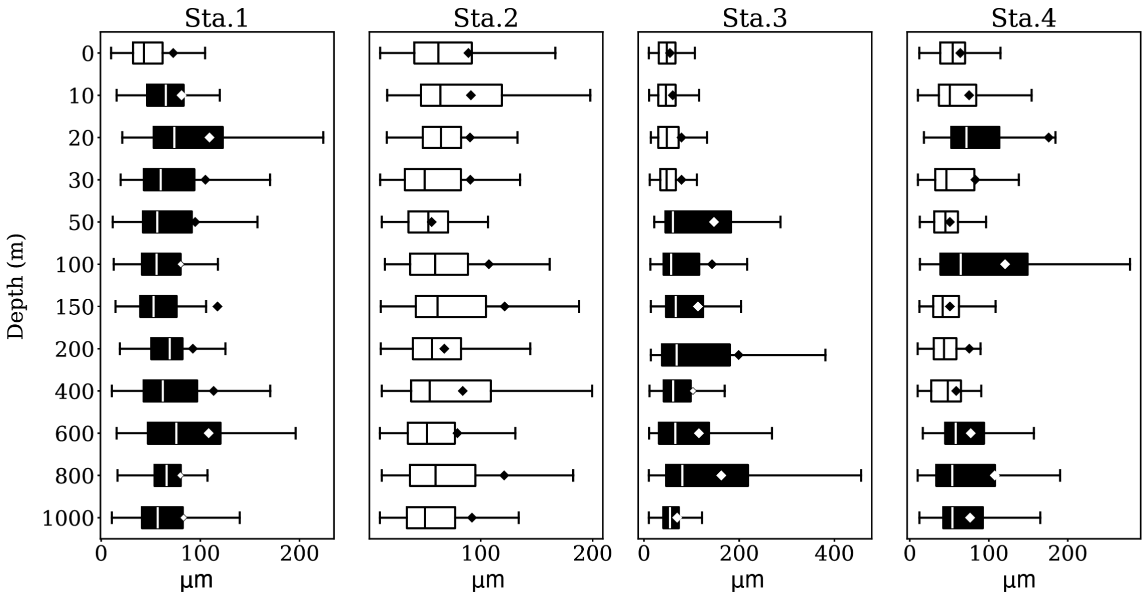

Fig. S6. Box plot of sampling depth versus SMP size at four stations. The black boxes indicate that sizes were significantly larger at that depth than at 0 m depth. Dots, bars within each box, the two ends of each box, and the two ends of each whisker represent the average, median, and 23rd/75th percentiles, and minimum/maximum of SMP sizes, respectively. Note that the sampling depth at Sta. 3 was 260 m, not 200 m.

S9. Percentages of fragments and fibers

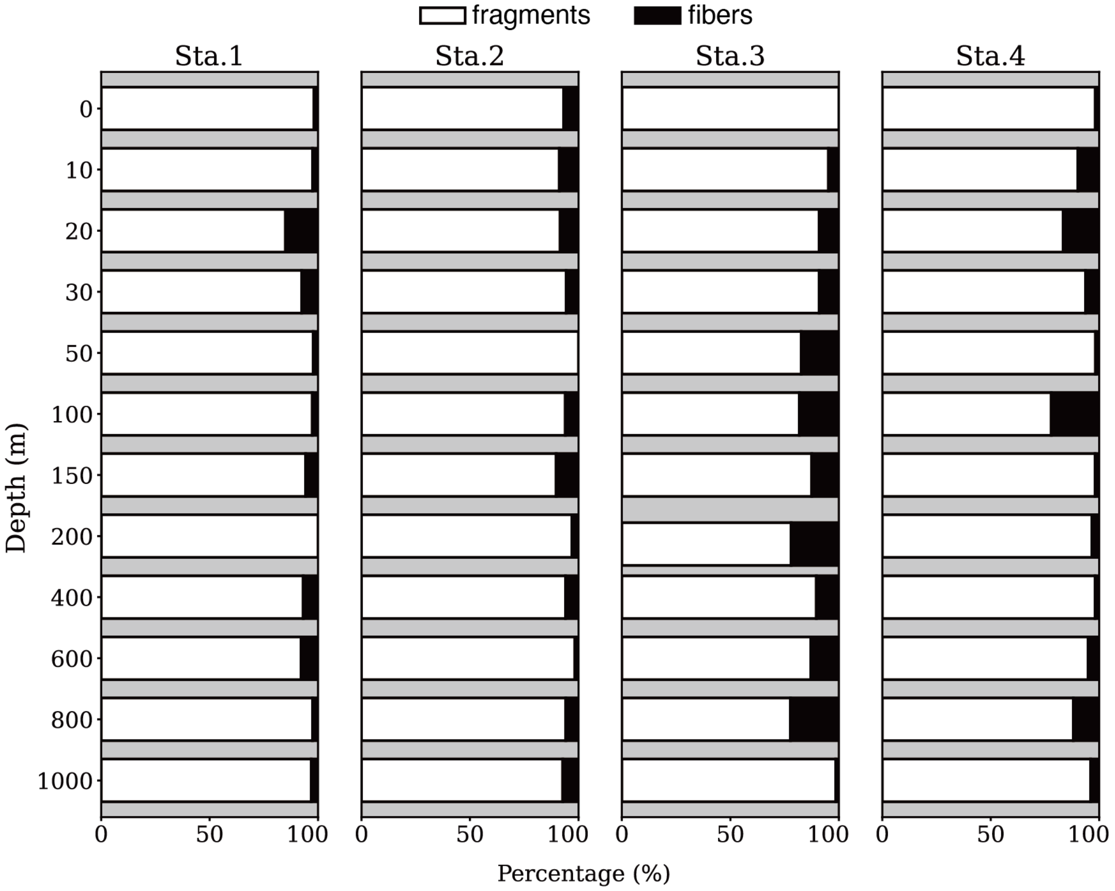

Fig. S7 Percentages of fragments (white) and fibers (black) at each depth at four stations. Note that the sampling depth at Sta. 3 was 260 m, not 200 m.

S10. Percentages of plastic polymers at each depth

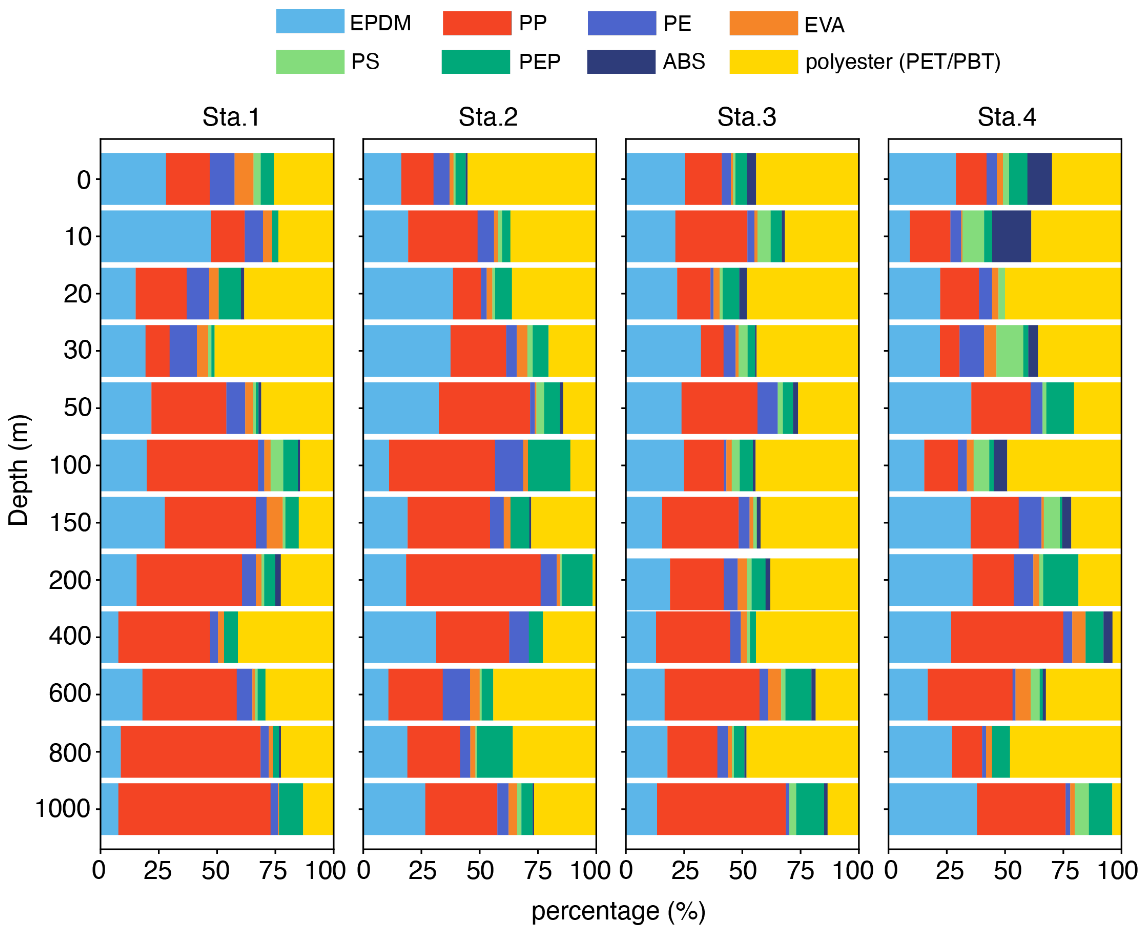

Fig. S8. Percentages of plastic polymers, indicated by color scale at the top, at each depth across four stations. Note that the sampling depth at Sta. 3 was 260 m, not 200 m.

S11. Correlation relationship between SMP concentration and salinity

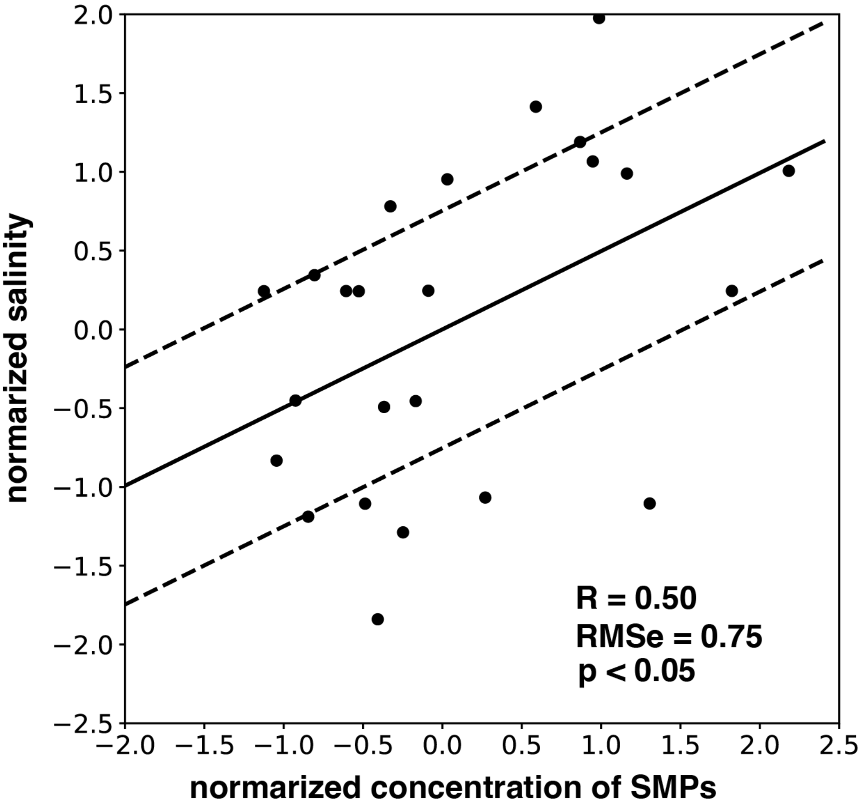

Fig. S9. Scatter plots and regression line between salinity and SMP concentrations, both normalized by their respective standard deviations. Dashed lines indicate the root mean square error from the regression line.

S12. MP concentrations at the sea surface

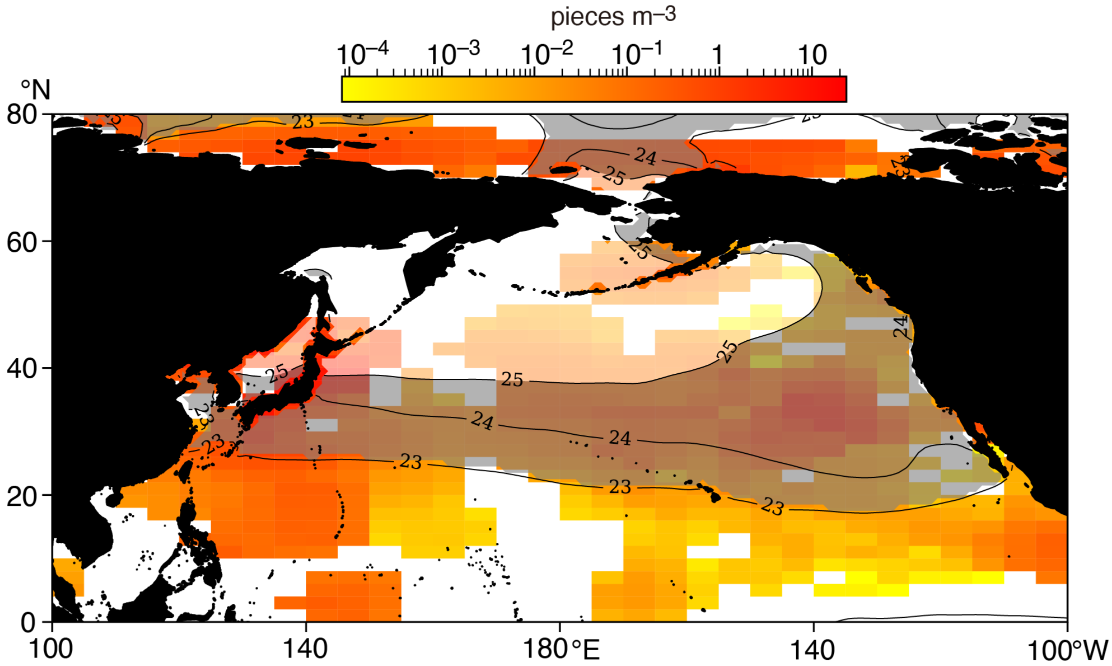

Fig. S10. MP concentrations at the sea surface, indicated by the color scale at the top. Concentrations (particle count per unit seawater volume) were downloaded from the Atlas of Ocean Microplastics (AOMI) website (https://aomi.env.go.jp) and averaged across the months of January to December. The climatological wind speed and wave height were used to compute averaged concentrations.[5] Stippling indicates the surface seawater density between 23σ_θ and 25σ_θ downloaded from the World Ocean Atlas (https://www.ncei.noaa.gov/products/world-ocean-atlas).

S13. Biofouling-Induced Changes in the Terminal Velocity of SMPs

We consider a single SMP particle that becomes progressively covered by algal biofilms, following the approach of Kooi et al. (2017).[6] For simplicity, the SMP is assumed to be a sphere with a diameter of δ_p (56 μm), corresponding to the median size of collected SMPs (subsection 3.2). Upon biofouling, the particle is similarly modeled as a sphere with an increased effective diameter δ (Fig. S11). The terminal velocity of biofouled SMP is estimated using Stokes' law for spherical bodies as $\delta^2(\rho_s - \rho^*)g/18\eta$, where $\delta, \rho_s, \rho^*, g$ and η, respectively, are effective diameter of biofouled particle, ambient seawater density (1025 kg m^{-3}), density of biofouled particle, gravitational acceleration, and dynamic viscosity of seawater (1.025×10^{-3} kg m^{-1} s^{-1}). The density of biofouled particle is calculated based on the volume-weighted average of the SMP core and surrounded biofilm (Fig. S11) as follows:

$$\rho^* = \frac{\left\{\frac{4}{3}\pi\left(\frac{\delta}{2}\right)^3 - \frac{4}{3}\pi\left(\frac{\delta_p}{2}\right)^3\right\}\rho_b + \frac{4}{3}\pi\left(\frac{\delta_p}{2}\right)^3\rho_p}{\frac{4}{3}\pi\left(\frac{\delta}{2}\right)^3} = \rho_b + \left(\rho_p - \rho_b\right)\left(\frac{\delta_p}{\delta}\right)^3,$$

where ρ_p and ρ_b are densities of SMP core (900 kg m^{-3}) and biofilms (1388 kg m^{-3}),[6] respectively. Terminal velocities were calculated for a range of effective diameters δ (0–120 μm), representing varying biofilm thickness. As shown in Fig. S11, SMPs coated with biofilms thicker than a few micrometers (approximately (62–56)/2 μm) begin to sink, exhibiting settling velocities comparable in magnitude to those of algal aggregates lacking SMP cores. Upward terminal velocities (>0 m day^{-1}) occur when $\rho_s > \rho^*$, and downward velocities when $\rho_s < \rho^*$.

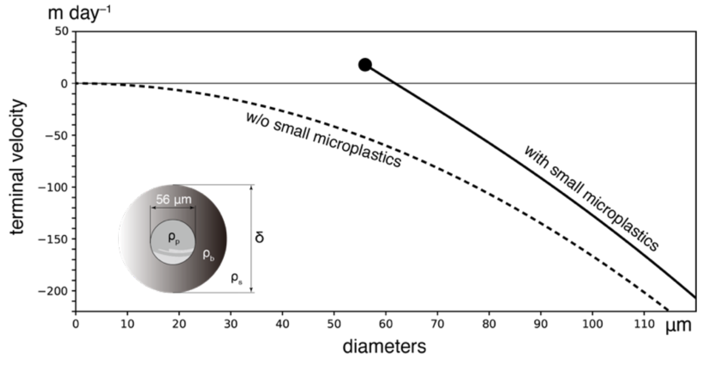

Fig. S11. Comparison of terminal velocities calculated with (solid curve) and without (dashed curve) a single SMP particle embedded in a single algal aggregate (bottom left). The upward velocity of a 56-μm SMP particle without biofouling is indicated by the filled circle.

Table S1. Field surveys of ocean small microplastics

Source	Location	Size range (μm)	Abundance (particles m^{-3})	Depth (m)	Equipment	Filtered volume
Desforges et al. (2014)[11,a]	eastern North Pacific	64.6–500 (63.6%[b])	1323	4.5	Intake water	-[f]
Enders et al. (2015)[12]	North Atlantic	11–100	13–501	3	Intake water	2.6 m^3
Poulain et al. (2019)[13]	North Atlantic	25–1000	3.8–60[c]	0	Neuston net (25 μm)	<540 m^3
Lorenz et al. (2019)[14]	Southern North Sea	<100 (86%)	0.1–211	0	Neuston net (100 μm)	15.51–51.30 m^3
Song et al. (2018)[15]	Korean coastal waters	50–300 (86%)	385–1720	3–58	Water pump	100L
Ding et al. (2019)[16]	South China Sea	<330 (67.2%)	134–8198	1–40	Water sampler	5L
Pabortsava et al. (2020)[17]	Atlantic	32–651	2272[d]	10–270	Water pump	507–1534L
Tekman et al. (2020)[18]	Arctic Ocean	11–200	0–1287	1–5350	Water pump	218–561L
Zhao et al. (2022)[19]	South Atlantic	20–321.2	0–244.3	10–5200	Water pump	440–1765L
Zhao et al. (2023)[20]	eastern North Pacific	25–100 (84.5%)	124–471	30–3700	Water pump	397–510L
Eo et al. (2021)[21]	Sea around Korea	20–5000	15–9400	15–2100	Water pump	200L
Present study	western North Pacific	20–300	2250–14375 (1575–10063)[e]	0–1000	Water sampler	16L (48L[g])

[a] See the reference list in the text for the article assigned to each reference number

[b] Percentage of MPs in the size range is shown in parentheses if the value was reported in the original paper. The abundance was computed by multiplying these percentages with the abundance presented in each paper.

[c] The abundance provided in particles/km^3 in the paper was converted through division by the potential seawater volume (10^6 m^2 × 0.1 m height of the net) that passed through the manta net.

[d] Sum of PE, PP, and PS particles collected in this study.

[e] 70% values excluding the suspected overestimates are shown in parentheses.

[f] not described.

[g] The abundance was discussed using SMPs in seawater volume integrated over three stations at each depth (i.e., 16L × 3; see Fig. 3).

Table S2. Survey site details.

Sta.	year/month/date	Longitude		Latitude		Water depth (m)
		Start	End	Start	End	
Sta.1	2022/11/16	140.469730	140.465085	29.998503	30.003282	2454
Sta.2	2022/11/19	140.997763	140.997207	19.999512	19.999640	4616
Sta.3	2022/11/22	141.005985	141.020495	3.541143	3.533113	3322
Sta.4	2022/12/4	128.999792	128.995202	26.811652	26.822117	2337

Table S3. Plastic polymers excluded in the present study

**Polymers to be excluded**	**Reason(s)**
Polyether sulfone	Use for Filter in Milli-Q water system
Polytetrafluoroethylene	Use for Membrane filter and holder lid
Polyvinyl chloride	Use for Niskin bottles
Polycarbonate	Use for Containers
Perfluoroalkoxy alkane	Use for Containers
Nylon (polyamide)	Contamination

Table S4. Abundances of SMPs by depth at four stations. Note that the sampling depth at Sta. 3 was 260 m, not 200 m.

Depth (m)	Concentration in pieces m^{-3}*				
	Sta.1	Sta.2	Sta.3	Sta.4	Average (particle count) ± standard deviation
0	10000	7250	6375	7125	7688 (123) ± 1376
10	4750	3563	14375	10813	8375 (134) ± 4423
20	4563	5188	9625	2250	5406 (86) ± 2670
30	9063	5500	9688	5938	7547 (120) ± 1848
50	5438	9250	2875	3688	5313 (85) ± 2455
100	6938	6188	5500	6500	6281 (100) ± 524
150	5438	8500	5565	8160	6916 (110) ± 1420
200	5250	8125	6250	7438	6766 (108) ± 1103
400	7313	5188	9625	3250	6344 (101) ± 2378
600	5563	7500	5684	4813	5890 (94) ± 988
800	7188	8563	9333	9000	8521 (136) ± 817
1000	13875	10313	4188	3125	7875 (126) ± 4418
				**Average**	**6910 (110)**

* Concentrations were computed as observed particle counts multiplied by 1000/16.

Reference

(1) Primpke, S.; Meyer, B.; Falcou-Préfol, M.; Schütte, W.; Gerdts, G. At second glance: The importance of strict quality control–A case study on microplastic in the Southern Ocean key species Antarctic krill, Euphausia superba. *Sci. Total Environ.*, **2024**, 918, 170618

(2) Turcotte, D. L. Fractals and fragmentation, *J. Geophy. Res*., **1986**, 91, 1921-1926.

(3) Kaandrop, M. K.; Dijkstra, H. A.; Van Seville, E. Modelling size distributions of marine plastics under the influence of continuous cascading fragmentation. *Environ. Res. Lett.*, **2021**, 16, 054075.

(4) Aoki, K.; Furue, R. A model for size distribution of marine microplastics: a statistical mechanics approach. PLoS ONE, 16, e0259781.

(5) Isobe, A.; Azuma, T.; Cordova, M. R.; Cózar, A.; Galgani, F.; Hagita, R.; Kanhai, L. D.; Imai, K.; Iwasaki, S.; Kako, S.; Kozlovskii, N.; Lusher, A.; Mason, S.; Michida, Y.; Mituhasi, T.; Morii, Y.; Mukai, T.; Popova, A.; Shimizu, K.; Tokai, T.; Uchida, K.; Yagi, M.; Zhang, W.. A multilevel dataset of microplastic abundance in the world's upper ocean and the Laurentian Great Lakes. *Microplastics and Nanoplastics*, **2021**, 1, 1-14.

(6) Kooi, M.; Van Nes, E. H.; Scheffer, M.; Koelmans, A. A. Ups and downs in the ocean: effect of biofouling on vertical transport of microplastics. *Environ. Sci. Tech*., **2017**, 51, 7963-7971.
